# The Impact of Non-Concentrated Storage on the Centrifugation Yield of *Microchloropsis gaditana*: A Pilot-Scale Study

**DOI:** 10.3390/life14010131

**Published:** 2024-01-17

**Authors:** Joran Verspreet, Floris Schoeters, Leen Bastiaens

**Affiliations:** 1 Flemish Institute for Technological Research (VITO), Boeretang 200, 2400 Mol, Belgium; leen.bastiaens@vito.be; 2 Radius, Thomas More University of Applied Sciences, 2440 Geel, Belgium; floris.schoeters@thomasmore.be

**Keywords:** *Nannochloropsis gaditana*, microalgae, wet preservation, respiration, cell rupture

## Abstract

Non-concentrated algae storage can bridge the period between algae harvesting and processing while avoiding the stress conditions associated with the concentration step required for concentrate storage. This study aimed to examine organic matter losses during the non-concentrated storage of *Microchloropsis gaditana* at pilot-scale. Algae cultures (400–500 L) were stored for up to 12 days either at an 8 °C target temperature or at 19 °C as the average temperature. The centrifugation yield of stored algal cultures decreased from day 5 or day 8 onwards for all storage conditions. After 12 days, the centrifugation yields were between 57% and 93% of the initial yields. Large differences in centrifugation yields were noted between the algae batches. The batch-to-batch difference outweighed the effect of storage temperature, and the highest yield loss was observed for the 8 °C cooled algae batch. The analysis of stored algae before and after centrifugation suggested that the decreasing yields were not related to respiration losses, but rather, the decreasing efficiency with which organic matter is collected during the centrifugation step.

## 1. Introduction

The interest in the commercial exploitation of microalgae is increasing, and microalgae production has tripled in the past five years [1]. Yet, the estimated global microalgae production (56,000 tons in 2019 [2]) remains rather low compared to that of traditional food or feed crops. A reduction in production costs is needed to further stimulate the development of the algae value chain. There are clear economies of scale, as a significant reduction in the production cost/kg algae is expected when algae cultivation volumes increase [3]. Synergies can be achieved by connecting a single biorefinery facility with cultivation facilities at multiple sites. When algae are collected at a central processing location, it becomes important to keep the algae under suitable storage conditions before and during their transport, ensuring quality maintenance. Also, when algae processing and cultivation are carried out right next to each other, temporary storage is often unavoidable in practice. Hence, a good understanding of the impact of temporary storage on algae quality is needed.

When algae waiting for processing needs to be stored for only a few days, wet, refrigerated storage is an attractive preservation method, though lipid and microbial stability are concerns [4]. The storage of algae concentrates obtained through centrifugation of the algae culture is the main focus in the algae literature [4,5]. The storage of algae concentrates (with organic matter levels around 100 g/L) obtained via this approach requires only minimal volumes to be refrigerated, and the potential transportation costs are minimized as well.

An alternative approach is to store the algae suspensions (about 1 g organic matter/L) without a preceding concentration step. Skipping the centrifugation step before storage prevents exposing the algae to shear stresses. The centrifugation step [6], but also the higher cell density [7], can be stressful for the algae and can, depending on the algal species, jeopardize algae viability during storage. Avoiding cell lysis may furthermore prevent the associated lipid degradation during storage [8]. Despite the possible advantages of storing algae without a preceding centrifugation step, this approach has received scant attention in the research literature. For *Porphyridum purpureum* (*Rhodophyta*), it was shown that non-concentrated storage can result in less formation of malodorous organic acids than during concentrated storage [9]. As long as *P. purpureum* suspensions are not concentrated, one can also avoid the formation of a viscous, difficult-to-process network, as seen for *P. purpureum* concentrates. A disadvantage of this non-concentrated storage approach is that large volumes of algae cultures must be stored. However, the pre-concentration of algae biomass (to 10–40 g OM/L) via low-shear technologies can offer a solution in this respect [10].

Another neglected aspect in this context is the effect of scale and storage volume. Storing, for example, cultures of a few 100 L instead of 1 L presents new challenges, such as ensuring proper mixing of the stored culture. It is therefore important to perform preservation tests on a sufficient scale, making the results relevant for storage in a commercial setting. Yet, no pilot-scale preservation studies have been published so far to our knowledge. Such experiments are obviously challenging as they require expensive infrastructure and large volumes of fresh algae.

Against this background, this study aimed to evaluate organic matter losses during non-concentrated, pilot-scale algae storage and the impact of storage temperature and algae batch thereon. The focus of this work was on organic matter losses during storage because (i) they directly impact the yield of the entire production process and (ii) they can be expected to depend on O_2_ exposure and scale effects. In the case of concentrate storage, a few studies have indicated that organic matter losses can be high and can amount to up to 35% after 14 days [9], and dry matter losses have reached 44% after 30 days [11]. However, no such data are available to our knowledge for non-concentrated storage, nor for pilot-scale storage. Algae were either stored at an average temperature of 19 °C or a lower temperature (target temperature 8 °C). Lower temperatures are expected to slow down microbial growth and unwanted biochemical reactions such as lipolysis [8]. Furthermore, 8 °C was previously shown to limit the formation of malodorous short-chain organic acids during the storage of concentrates of *Microchloropsis gaditana*, formerly known as *Nannochloropsis gaditana* (Eustigmatophyceae) [12], and has the added benefit of limiting cooling costs compared to, for instance, 4 °C cooling. *M. gaditana* was used for this study. *Microchloropsis* sp. are the most important species for feed applications in Europe, and are, after *Chlorella* sp. (Chlorophyta) and *Spirulina* (Cyanobacteria), the main microalgae produced in Europe [13]. This work is a first step towards scaling up the preservation of non-concentrated algae suspensions.

## 2. Materials and Methods

### 2.1. Pilot-Scale Algae Storage: Algae Cultivation and Storage Conditions

*Microchloropsis gaditana* was cultivated in a greenhouse at the Sunbuilt facility (Geel, Belgium) in a 1500 L tubular photobioreactor. A permeate obtained through membrane filtration of an open-pond algae culture grown on process water from a demineralization unit formed the basis for the algae growth medium. Nitrogen, phosphorus, potassium, and microelements were added as described before [14], except for NaCl, which was not added in the present study because of the presence of salt in the process water. Cultures were inspected microscopically for the absence of grazers and contaminating algae species (Supplementary Figure S1). The turbidity in the bioreactor was continuously monitored and algae were always harvested within one day once they reached a turbidity of 800 NTU (nephelometric turbidity units). Nephelometry enables the continuous monitoring of dry matter (DM) levels, which were also verified by bi-weekly sampling and oven drying-based DM analysis [15]. For each storage test, between 400 and 500 L of fresh culture was pumped into a 2000 L stainless steel, double-walled storage vessel. To equalize the initial algae concentration and culture volume in the storage vessel, small volumes (<100 L) of membrane filtration permeate were added so that the t_0_ volume (Supplementary Figure S2) and the organic matter concentrations (3–4 g organic matter/L) were similar for all tests. The algae culture was stirred continuously during storage, and the temperature was controlled in most experiments by an outer layer of coolant around the tank. More details on the storage tank geometry and dimensions can be found in Supplementary Figure S3. During storage and before algae sampling, O_2_ levels were measured using a multi340i portable meter (WTW xylem, Washington, WA, USA) and a VWR OXY 11-DO Sensor (VWR, Leuven, Belgium) at the top of the stored culture for most time points and the first two storage tests (Supplemental Table S2). Prior to algae sampling, the algae suspension was pumped out of the tank and immediately back into the tank to ensure the homogeneity of the suspension and to allow proper sampling. Next, aliquots were centrifuged by a batch centrifuge (10 min, 15,000× *g*, Sorvall LYNX 6000, Thermo Scientific, Waltham, MA, USA). Centrifugation was performed at least in duplicate, and the centrifugation yield (g organic matter in pellet/L algae culture) was determined by weighing the initial algae and final pellets and determining their dry and organic matter contents. For stored algae, the relative centrifugation yield was calculated as follows:

$$Relative\;centrifugation\;yield\;\left(\%\right)\;=\;\frac{yield\;after\;storage\;({g\;organic\;matter/L\;algae\;culture)}_{t}}{initial\;yield\;({g\;organic\;matter/L\;algae\;culture)}_{t_{0}}}$$

The obtained algae pellets were stored at −25 °C for later biochemical analyses (Section 2.3).

### 2.2. Biochemical Analyses

Dry matter content was determined by weighing the samples before and after overnight drying at 105 °C. The organic matter content was assessed by weighing the dried samples before and after 4 h incubation at 550 °C. The lipid and carbohydrate content of the algal pellets obtained by centrifugation was evaluated after freeze-drying of the algal pellets. The total lipid content was determined by chloroform:methanol extraction, as described by Ryckebosch et al. [16]. For the total carbohydrate analysis, two-step H_2_SO_4_ hydrolysis was performed, and the released monosaccharides and uronic acids were quantified by high-performance anion exchange chromatography with pulsed amperometric detection, as described before [9]. The total carbohydrate level was estimated by summing the monosaccharide and uronic acid levels, each corrected for water uptake during hydrolysis.

### 2.3. Statistical Analysis

Statistica version 12 (Dell Inc., Tulsa, OK, USA, 2015) was used for statistical analyses with 5% as the significance threshold level. One-way ANOVA was used to assess whether algae quality parameters differed among algae with different storage times. In the case of a positive omnibus test, a Tukey multiple comparison test was carried out.

## 3. Results and Discussion

### Pilot-Scale Algae Storage

**Figure 1:**
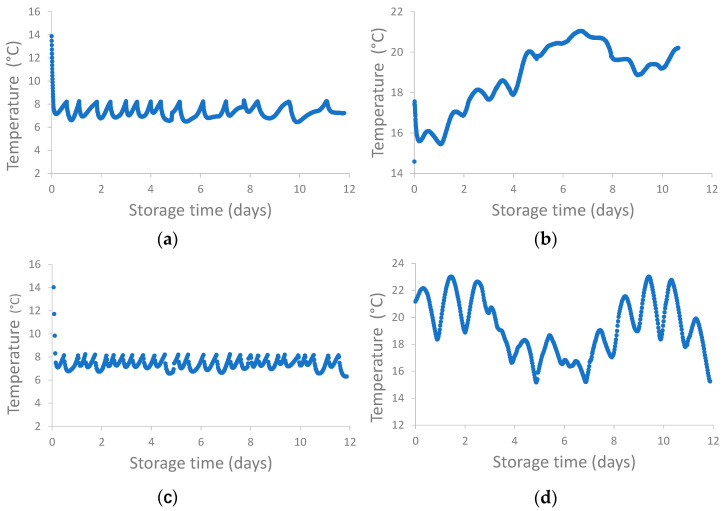
Temperature profiles during pilot preservation tests with (**a**) algae batch 1 and 8 °C target temperature; (**b**) algae batch 2 and 19 °C average temperature; (**c**) algae batch 3 and 8 °C target temperature; and (**d**) algae batch 3 without temperature control.

First, two consecutive storage tests were performed in a temperature-controlled storage vessel with 8 °C as the target temperature (algae batch 1, harvested on 20 April 2022) and one with 19 °C as the average temperature (algae batch 2, harvested on 5 May 2022). Next, another test was performed to evaluate the temperature effect in an algae-batch-independent way. Fresh biomass (algae batch 3, harvested on 2 June 2022) was split and either stored in the temperature-controlled storage vessel with 8 °C as the target temperature or stored in a similar storage vessel at ambient temperature without temperature control. Because the temperature-controlled storage vessel was only able to cool, the temperature was still variable to some extent (Figure 1).

When the target temperature was set at 8 °C, the average temperatures were similar and were 7.3 ± 0.5 °C (algae batch 1, Figure 1a) and 7.5 ± 1.1 °C (algae batch 3, Figure 1c). The average temperatures of the two other experiments were also similar, i.e., 18.9 ± 1.6 °C (Figure 1b) and 19.2 ± 2.1 °C (Figure 1d) for algae batches 2 and batch 3, respectively. Also, the volumes of the stored cultures were similar (Supplementary Figure S2). Note that storing algae batch 3 without temperature control resulted in relatively large temperature differences every 24 h due to the day vs. night temperature differences.

**Figure 2:**
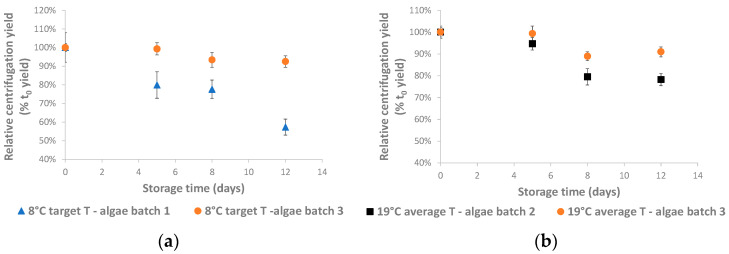
Relative centrifugation yields during pilot-scale storage (**a**) with 8 °C as the target temperature and (**b**) 19 °C as the average temperature. Absolute values are shown in Supplementary Table S1, including the results of a one-way ANOVA analysis thereon. Details about the temperature of the algae suspension as a function of storage time can be found in Figure 1.

After the non-concentrated storage, aliquots of the stored culture were concentrated by batch centrifugation, and the centrifugation yield was determined. the Absolute yield values (g organic matter in pellet/L stored algae culture) are given in Supplementary Table S1, while the relative centrifugation yields (%, relative to the t_0_ yield) are plotted in Figure 2.

The centrifugation yield was clearly affected by storage time in each storage test (*p* < 0.01) and decreased significantly starting from day 5 or day 8 onwards (Figure 2 and Supplementary Table S1). Surprisingly, the lowest yield values were observed after low-temperature storage (8 °C storage temperature—algae batch 1) and not with 19 °C as the average storage temperature. Additionally, clear batch-to-batch differences were noted, especially with 8 °C as the target temperature (Figure 2a).

One explanation for the decreasing centrifugation yields could be a loss of biomass during storage due to respiration. Accordingly, the oxygen levels measured at the top of the culture in the storage vessels correlated to some extent with the relative centrifugation yields (Supplementary Figure S4). In addition to respiration processes, fermentation processes can also result in the degradation of organic compounds and their conversion into volatile compounds, and hence, dry matter loss, as seen for *Monoraphidium* sp. biomass [17]. Yet, this explanation or that of respiration losses was not supported by the analysis of the organic matter levels in the stored cultures before centrifugation (Table 1).

Indeed, the organic matter levels after 12 days of storage were not significantly different from those at the start for three out of the four storage experiments, including for the test where the highest relative decrease in centrifugation yield was observed (algae batch 1 and 8 °C target temperature). Only for the storage of algae batch 3 and the 8 °C target temperature was a limited decrease in organic matter observed. This suggests that respiration losses have little or no impact on centrifugation yields. Yet, a note of caution is due here, since the standard deviations of the measured organic matter levels in the algae cultures (up to 0.14 g/L, Table 1) are rather high compared to the differences in the centrifugation yields (on average, 0.28 g organic matter pellet/L culture in algae batch 1 after 12 days), hampering the detection of organic matter level differences that could explain decreasing centrifugation yields. Therefore, the composition of the pellets obtained after centrifugation of the retained biomass was analyzed (Table 2), as this might provide additional experimental evidence for the occurrence or lack of respiration losses [18].

For the algae batch with the highest decrease in centrifugation yield (i.e., 43% relative decrease for algae batch 1 and 8 °C target storage temperature), the lipid (*p* = 0.86) and carbohydrate levels (*p* = 0.48) in the obtained pellets were not affected by storage time. For algae batch 2 stored at 19 °C, the carbohydrate levels decreased slightly during storage (Table 2). For algae batch 3, the lipid levels were stable, while the carbohydrate levels changed slightly (Table 2). The degradation of reserve carbohydrates [7,9,19,20] or lipids [18] was sometimes observed in previous storage studies. However, lipid levels did not decrease in this study, and no decrease in carbohydrate levels, or only a very small decrease, compared to the loss in centrifugation yield was observed. Consequently, the selective degradation of carbohydrates or lipids does not seem to be the main cause for lower centrifugation yields.

An alternative explanation for the decreasing centrifugation yields seen in Figure 2 is a decrease in the centrifugation recovery efficiency, or the amount of organic matter precipitated during centrifugation. This may occur due to increased cellular excretions or a gradual increase in cell lysis, which releases soluble intracellular material. This was previously shown to already begin at the end of the cultivation period in the case of *Tetradesmus lagerheimii* (formerly *Scenedesmus acuminatus*) [21], *Chromochloris zofingiensis* (formerly *Chlorella zofingiensis*) [22], and *Dunaliella salina* (*Chlorophyta*) [23], and can be expected to become more prominent during storage. Indeed, cell lysis was cited to explain the appearance of extracellular chlorophyll and algal DNA during the storage of *Chlamydomonas nivalis* concentrates obtained through batch centrifugation [24], and to explain the strong increase in cell permeability during the storage of *Nostoc flagelliforme* (*Cyanobacteria*) concentrates [25]. The extent to which shear stresses lead to cell rupture depends on the species used [26] and especially on cell wall rigidity [8], which, in turn, may depend on environmental conditions such as medium salinity [27]. *Microchloropsis* sp. cell walls and also those from *Chlorella* sp. are considered rigid cell wall sp., while *Arthrospira platensis* (*Cyanobacteria*) and *Porphyridium purpureum* (formerly *Porphyridium cruentum*) (*Rhodophyta*) have fragile cell walls [28]. Another possible cause for decreasing centrifugation efficiency is a changing cell surface electrostatic charge, which can be affected by both algae activities and the surrounding environment [29].

In conclusion, there are several possible mechanisms by which storage can affect centrifugation recovery efficiency, and for one of them, the increased release of algae organic matter in the extracellular phase, a link with algae storage has been shown before. Although conclusive evidence is missing, decreasing centrifugation recovery efficiency is a credible explanation for the decrease in centrifugation yields observed in the present study, especially since our data ruled out the alternative explanation, the degradation of reserve compounds prior to centrifugation.

Based on previous studies, it was expected that cooling would favor algae preservation. Lower temperatures (4 °C) promoted the cell viability of Chaetoceros calcitrans (Bacillariophyta) concentrates compared to 27 °C storage [30]. Cooled storage (4 °C) also enabled the long-term preservation (40 days) of *Tetraselmsis suecica* (*Chlorophyta*) (4 g/L) with good retention of cell viability in terms of cell motility and photosynthetic activity [7]. However, the current study differs from previous research in focusing on the centrifugation yields of dilute cultures, where centrifugation takes place after storage. Centrifugation, and the associated shear and hydrodynamic stress [23], might affect cell integrity more when applied on stored algae than on fresh algae. Indeed, stored algae might have been weakened by the stressful storage conditions they experienced, such as the long periods of darkness and lack of nutrient supply. In any case, no clear temperature effect could be observed here, while there were strong differences between algae batches stored at the same temperatures.

The surprisingly large differences between batches in this study may stem from slight differences in growth conditions or stress exposure levels during growth. The different batches were cultivated sequentially in spring, with changing factors like light exposure and temperature, which are typically controlled in lab-scale tests.

More research is required to understand why there were such large batch-to-batch differences. Special attention should be paid to pre-harvest factors such as the nutritional status of the algae or exposure to stress during the growing period. A combination of both laboratory-scale and pilot-scale experiments is recommended. Indeed, growth and storage experiments can be performed under controlled conditions at laboratory scale and enable the simultaneous testing of multiple factors (number of growth and harvest cycles, nutrient status, climatic conditions, microbiota, etc.) and multiple factor levels with one algae batch. Pilot-scale testing is, however, also needed, as this scale is characterized by its own boundary conditions closer to those of an industrial-scale process.

## 4. Conclusions

This study demonstrated that centrifugation yields can decrease significantly during non-concentrated algae storage, and this decrease can have a strong impact on the yield of the entire algae production process. Large differences were observed between batches in terms of centrifugation yield loss. Moreover, cooling the algal culture could not prevent centrifugation yield loss, at least for the first algae batch. A decrease in centrifugation efficiency, possibly due to increased cell rupture, seems to be the most likely cause for decreasing centrifugation yields.

## Figures and Tables

**Table 1:** Organic matter concentrations during pilot-scale storage. Lowercase letters (a,b) denote significant differences; values within one column and within one storage test that are not denoted by a common letter are significantly different.

Storage Time (Days)	Organic Matter (g/L)	Organic Matter Retained (% of t_0_ Organic Matter)
8 °C target T—algae batch 1 *		
0	2.77 ± 0.08 (a)	100.0% ± 2.8% (a)
5	2.84 ± 0.11 (a)	102.4% ± 4.1% (a)
8	2.85 ± 0.08 (a)	103.0% ± 3.0% (a)
12	2.76 ± 0.03 (a)	99.7% ± 1.2% (a)
8 °C target T—algae batch 3 *		
0	3.18 ± 0.07 (a)	100.0% ± 2.1% (a)
5	3.10 ± 0.08 (ab)	97.5% ± 2.4% (ab)
8	3.05 ± 0.06 (b)	96.0% ± 1.9% (b)
12	3.03 ± 0.02 (b)	95.4% ± 0.6% (b)
19 °C average T—algae batch 2 *		
0	4.02 ± 0.02 (a)	100.0% ± 0.6% (a)
5	3.99 ± 0.14 (a)	99.1% ± 3.6% (a)
8	3.85 ± 0.05 (a)	95.7% ± 1.3% (a)
12	3.92 ± 0.08 (a)	97.6% ± 2.1% (a)
19 °C average T—algae batch 3 *		
0	3.18 ± 0.07 (a)	100.0% ± 2.1% (a)
5	3.09 ± 0.04 (ab)	97.2% ± 1.4% (ab)
8	3.03 ± 0.04 (b)	95.4% ± 1.1% (b)
12	3.11 ± 0.04 (ab)	97.8% ± 1.2% (ab)

* Figure 1 illustrates algae suspension temperature during storage in detail.

**Table 2:** Lipid and carbohydrate levels in the biomass pellets obtained through centrifugation of the stored cultures. Lowercase letters (a–c) denote significant differences; values within one column and within one storage test that are not denoted by a common letter are significantly different.

Storage Time (Days)	Lipid Level in Pellet (% dm)		Carbohydrates * in Pellet (% dm)
8 °C target T—algae batch 1 **			
0	22.1 ± 0.5 (a)		14.6 ± 0.2 (a)
5	21.9 ± 1.1 (a)		16.2 ± 2.6 (a)
8	22.1 ± 0.6 (a)		14.2 ± 0.1 (a)
12	21.5 ± 0.4 (a)		14.3 ± 0.0 (a)
8 °C target T—algae batch 3 **			
0		18.3 ± 0.1 (a)	23.2 ± 0.4 (a)
5		18.3 ± 0.5 (a)	24.1 ± 0.7 (ab)
8		19.6 ± 0.3 (a)	23.2 ± 0.1 (a)
12		18.9 ± 0.5 (a)	24.8 ± 1.0 (ab)
19 °C average T—algae batch 2 **			
0	23.5 ± 0.3 (ab)		17.8 ± 0.3 (a)
5	22.9 ± 1.7 (b)		16.7 ± 0.1 (b)
8	24.7 ± 0.1 (ab)		16.1 ± 0.1 (bc)
12	26.5 ± 0.1 (a)		15.8 ± 0.3 (c)
19 °C average T—algae batch 3 **			
0	18.3 ± 0.1 (a)		23.1 ± 0.4 (a)
5	20.2 ± 0.0 (a)		21.9 ± 0.1 (b)
8	20.5 ± 0.4 (a)		21.8 ± 0.5 (b)
12	22.8 ± 2.4 (a)		22.6 ± 0.4 (ab)

* Carbohydrate levels were estimated by summing monosaccharide and uronic acid levels released after acid hydrolysis. ** Figure 1 illustrates algae suspension temperature during storage in detail.

## Data Availability

The raw data supporting the conclusions of this article will be made available by the authors on request.

## Supplementary Materials

The following supporting information can be downloaded at: https://www.mdpi.com/article/10.3390/life14010131/s1, Figure S1: Microscopy images of the *M. gaditana* cultures immediately before the start of the pilot-scale storage test; Figure S2: Volume profiles of algae cultures during the pilot-scale storage tests; Figure S3: Storage tank dimensions and geometry; Figure S4: Relative centrifugation yields of stored algae cultures as a function of O_2_ levels in the storage tanks; Table S1: Centrifugation yields during pilot-scale preservation; Table S2: O_2_ levels in the algae suspensions of the first two storage tests.
