# Supplementary material for: The Impact of Non-Concentrated Storage on the Centrifugation Yield of Microchloropsis gaditana: A Pilot-Scale Study
Source: Life (Basel). 2024 Jan 17;14(1):131. doi: 10.3390/life14010131 (PMC10821389; doi:10.3390/life14010131)
Supplement: Supplementary file 1 [file life-14-00131-s001.zip › life-2705324-supplementary.pdf]

The impact of non-concentrated storage on the centrifugation yield of *Microchloropsis gaditana*: A pilot-scale study

Joran Verspreet[1,*], Floris Schoeters[2], and Leen Bastiaens[1]

1 Flemish Institute for Technological Research (VITO), Boeretang 200, 2400 Mol
2 Radius, Thomas More University of Applied Sciences, Geel, Belgium

Supplementary information

Supplementary Figure S1

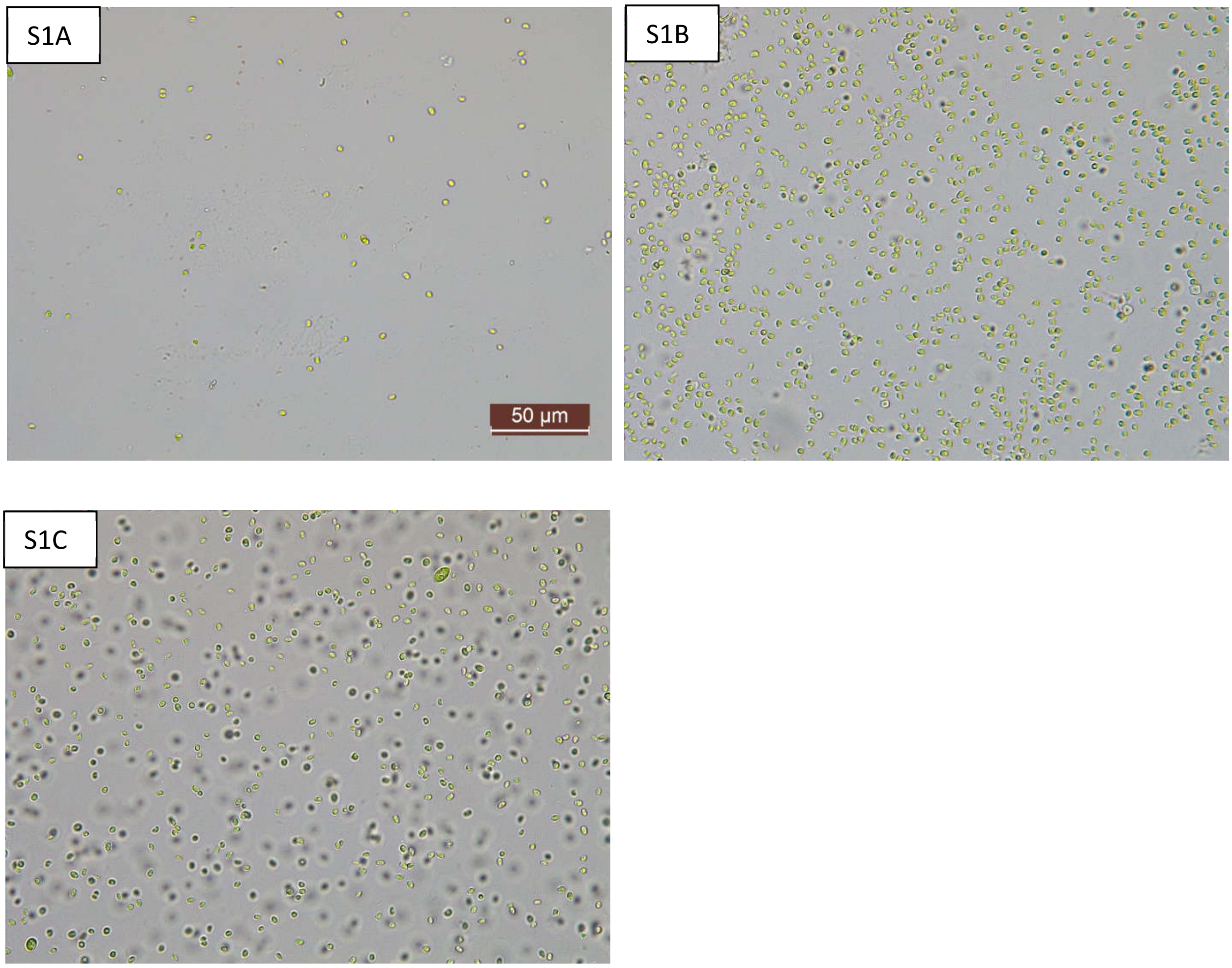

Supplementary Figure S1. *N. gaditana batch 1, one day before the start of the storage test (S1A), N. gaditana batch 2, two days before the start of the storage test (S1B), and N. gaditana batch 3, one day before the start of the storage test (S1C). Note that these pictures do not give a representative view of cell concentrations. The reader is referred to Table 1 (showing initial organic matter levels) to assess the algae concentrations in the different batches.*

Supplementary Figure S2

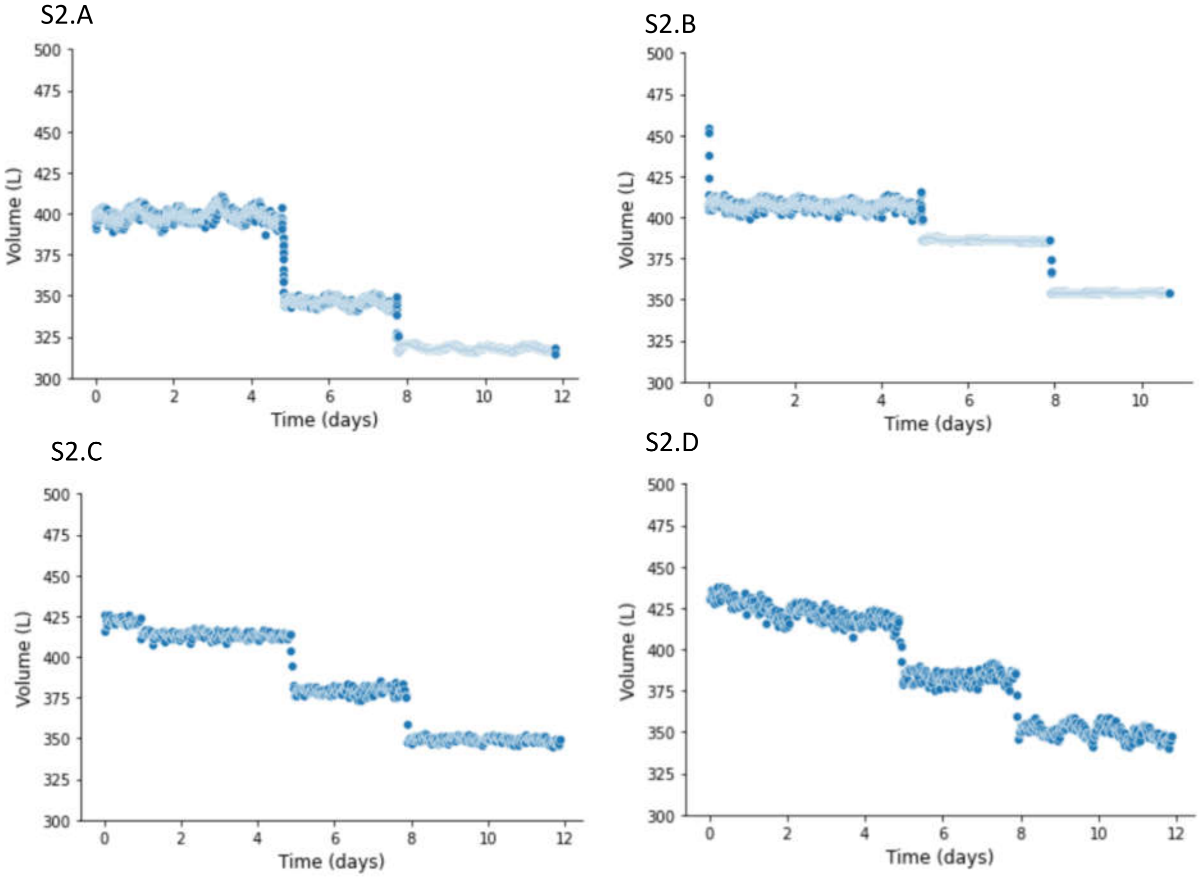

Supplementary Figure S2. *Volume profiles for pilot preservation tests with algae batch 1 and target temperature at 8°C (S2.A), algae batch 2 and target temperature at 20°C (S2.B), algae batch 3 and target temperature at 8°C (S2.C), and algae batch 3 and no temperature control (S2.D).*

Supplementary Figure S3

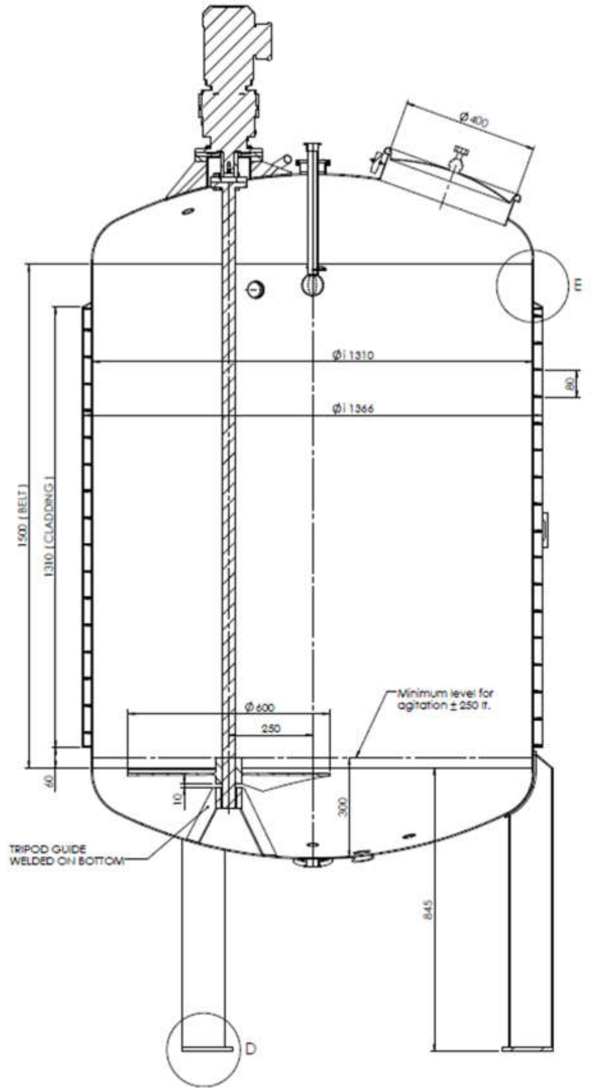

Supplementary Figure S3. *Storage tank dimensions and geometry.*

Supplementary Figure S4

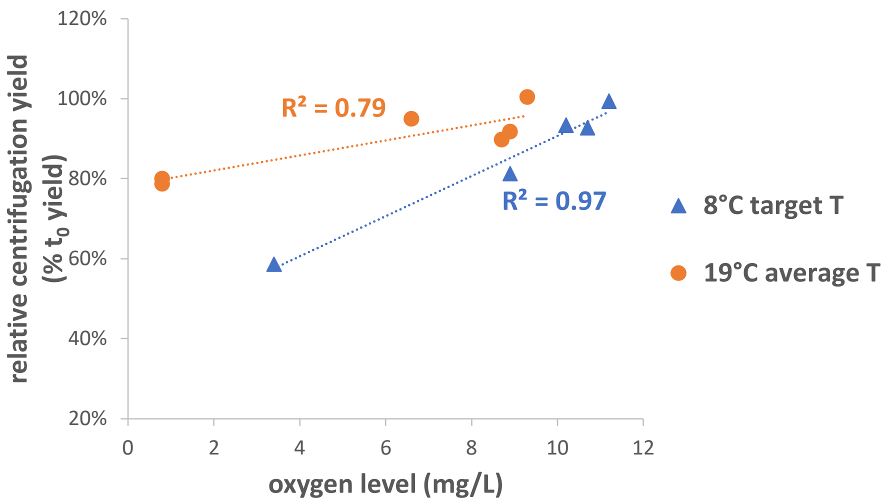

***Supplementary FigureS 4**. Relative centrifugation yields of stored algae cultures in function of O_2 levels in the storage tanks. Due to practical limitations, the O_2 levels were measured at the top layer of the algae suspension, and are probably higher than those at the bottom layers.*

Supplementary Table S1. Centrifugation yields during pilot-scale preservation. Lowercase letters (a, b, c) denote significant differences; values within one column and within one storage test that are not denoted by a common letter, are significantly different.

Storage time (days)	Centrifugation yield (g org matter pellet/L culture)	Relative centrifugation yield (% t_0 yield)	Storage time (days)	Centrifugation yield (g org matter pellet/L culture)	Relative centrifugation yield (% t_0 yield)
**8°C target T - algae batch 1***			**19°C average T - algae batch 2***		
0	0.66 ± 0.04 (a)	100.0 ± 7.9% (a)	0	0.70 ± 0.01 (a)	100.0 ± 2.8% (a)
5	0.53 ± 0.04 (b)	79.9 ± 7.1% (b)	5	0.67 ± 0.02 (b)	94.7 ± 2.9% (b)
8	0.51 ± 0.02 (b)	77.6 ± 5.0% (b)	8	0.56 ± 0.02 (c)	79.5 ± 3.7% (c)
12	0.38 ± 0.02 (c)	57.3 ± 4.3% (c)	12	0.55 ± 0.02 (c)	78.3 ± 2.8% (c)
**8°C target T - algae batch 3***			**19°C average T - algae batch 3***		
0	0.66 ± 0.01 (a)	100.0 ± 2.2% (a)	0	0.66 ± 0.01 (a)	100.0 ± 2.2% (a)
5	0.65 ± 0.02 (a)	99.3 ± 3.2% (a)	5	0.65 ± 0.02 (a)	99.3 ± 3.5% (a)
8	0.61 ± 0.02 (b)	93.4 ± 4.0% (b)	8	0.58 ± 0.01 (b)	88.9 ± 2.1% (b)
12	0.61 ± 0.02 (b)	92.5 ± 3.1% (b)	12	0.60 ± 0.01 (b)	91.0 ± 2.3% (b)

*Figure 1 in the main text illustrates algae suspension temperature during storage in detail.

Supplementary Table S2. O_2 levels in the algae suspensions of the first two storage tests. Due to practical limitations, the O_2 levels were measured at the top layer of the algae suspension, and are probably higher than those at the bottom layers.

Storage time (days)	O_2 levels (mg/L)	Storage time (days)	O_2 levels (mg/L)
**8°C target T - algae batch 1**		**19°C average T - algae batch 2***	
0	4.9 ± 0.1	0	not measured
5	8.9 ± 0.1	5	6.7 ± 0.1
8	not measured	8	0.9 ± 0.1
12	3.4 ± 0.1	12	0.9 ± 0.1
